# The Principal at Risk: Stress and Organizing Mindfulness in the School Context

**DOI:** 10.3390/ijerph17176318

**Published:** 2020-08-31

**Authors:** Pierluigi Diotaiuti, Stefania Mancone, Fernando Bellizzi, Giuseppe Valente

**Affiliations:** Department of Human Sciences, Society and Health, University of Cassino and Southern Lazio, 03043 Cassino, Italy; s.mancone@unicas.it (S.M.); fernando.bellizzi@unicas.it (F.B.); giuseppe.valente@unicas.it (G.V.)

**Keywords:** school principal, chronic stress, work discomfort, organizing mindfulness, health

## Abstract

*Background:* In recent years the role of school principals is becoming increasingly complex and responsible. *Methods:* This study was voluntarily attended by 419 Italian school principals who were administered the Psychological Stress Measurement (MSP), Mindfulness Organizing Scale (MOS), Polychronic-Monochronic Tendency Scale (PMTS), and the Scale of Emotions at Work (SEW). *Results:* The study has produced a path analysis model in which the relationships between the main predictors of principals’ work discomfort were explained. The effect of depressive anxiety on perceived discomfort (ß = 0.517) found a protective mediator in the mindfulness component that recognizes the sharing as a fundamental operational tool (ß = −0.206), while an increasing sense of effort and confusion could significantly amplify the experience of psychological discomfort associated with the exercise of school leadership (ß = 0.254). *Conclusions:* The model developed in this study suggests that focusing on organizing mindfulness can be a valuable guideline for interventions.

## 1. Introduction

Research on work-related stress has long identified the school as one of the risk environments, and various studies and publications deal with the stress and burnout of school workers. Most of the work produced devotes attention to teachers, and there are few reflections on the psychophysical resistance of the principal, especially considering all the legislative and organizational changes that have taken place [1,2]. These continuous changes, necessary in a rapidly changing society (well described by the “liquid society” metaphor coined by Zygmunt Bauman) become a cause of work-related stress for all school staff, especially principals. The ongoing reform of the education system in Italy involves various professionals and individuals who have a role in the complex world of school: Principal (DG—*Dirigente scolastico* in Italian); Director of General and Administrative Services (DSGA—it. *Direttore dei Servizi Generali e Amministrativi*), all Administrative, Technical and Auxiliary Staff (ATA—it. *personale Amministrativo, Tecnico e Ausiliario*), teachers, students and their parents (or parental figures). Each of these players must adapt to regulatory changes, both individually and collectively, with time dictated by political choices, which can be both national and supranational; the educational system, through the schools operating on the territory and the people who effectively act and put into practice the regulatory frameworks decided by the government institutions, must follow and pursue the changes in a short time period, often far from the actual physiological timing that the institution can implement.

The principal is an intermediate figure and a link between the institutional organizational framework, represented by the State, the Regions, and the Municipalities, and the framework of school individuals, represented by the teaching staff, ATA staff, parents and students. Many of the skills and competences required of professional staff, but also students, are not part of the training acquired over time and especially put into practice and enriched by personal experimentation and experience, so that even people with a ten-year work experience are forced to become new students learning subjects and skills hitherto untested, and therefore they are unaware of how gifted, capable and effective they are in resolving tasks [3,4]. The role of principal requires new skills and competences to meet the requirements and objectives defined by the proliferation of school reforms, regulations, and cultural changes [3,5,6].

Extensive research in various countries has already focused on principal burnout [7,8,9,10,11,12,13,14]. As Sari [8] notes, there may be curiosity and desire to learn and experience new skills and abilities, but the stress related to possible administrative errors and criminal consequences of errors or omissions, or low performance in failing to achieve results, remains high, with emotional drain and possible burnout consequences. Moreover, implicitly, the principal may also experience the feeling that his own cultural and cognitive skills, as well as tools and resources, have become obsolete or are no longer suitable for current needs, thus increasing personal insecurity [15]. These elements are sources of elevated stress levels in principals, because they become the point of reference and the catalyst of the stress of all professional and non-professional figures. Not only must the principal solve his/her own problems, but he/she must also deal with those of his/her subordinates, in most cases without having adequate leadership experience and training. Some countries have experienced a lack of candidates for the role of principal or an increase in resignations, because the teachers know they are not actually capable of fulfilling all the requirements the role calls for [16,17,18]. The principal experiences a drastic reduction in his/her educational and pedagogical function and must acquire technical, administrative, cognitive and what we could call management engineering skills: As a principal he/she becomes responsible for the safety of staff and students and a manager of economic resources, with budgetary obligations. To carry out these tasks, he/she must have medical-legal and managerial skills which the teacher who is promoted to the role of principal does not possess. Moreover, the change in commitments, as well as responsibilities, can be a source of great stress and a health risk, since the individual might not be able to manage, but above all to identify, the point where he must ask for help and activate a course of treatment. This applies both at the individual level and as the head of the safety and health of subordinate staff, having to decide when it is appropriate to subject a member of staff to a medical examination, especially when the latter does not want to or cannot deal with personal problems.

The principal is not alone and employs ATA and DSGA personnel, but as the person responsible for their work, he/she still must have the ability to evaluate and correct any errors. The above concerns only the legal-administrative part and the school is viewed as a business that must be run efficiently [19]. In addition to this, however, there is the management of the team in the areas of human resources and coordination. Principals can contribute to shaping the school climate as they should promote and support students and teachers [20]. This involves managing aspects related to students, social change and the management of a multi-ethnic society in continuous evolution, with changes in customs and processes [21,22].

For example, the new migratory flows have led some educational institutions to deal with the task of managing adult “students”, independent adults and adults with a “different” culture, who are normally obliged to attend school: However, this type of “student” requires cultural and relational skills and management skills that the school staff may not have.

The aim of this research is to draw attention to the figure of the principal as a person at risk of burnout and work-related stress for both exploratory, diagnostic and preventive purposes. The principal has to focus on the limit between being engaged in school life and avoiding workaholism, in order to live in a state of health and well-being [23]. Chronic stress stimulates negative behaviors and thoughts and problems related to emotions, feelings and physical health that can hinder effective school administration. According to scholars as Langer [24], Davidson et al. [25], Vogus and Sutcliffe [26], Weick and Putnam [27], and Weick and Sutcliffe [28], many positive benefits can be associated with individual and organizing mindfulness, such as health improvement, stress reduction, increased creativity, and less risk of burnout. More specifically Hoy et al. [29] define “school mindfulness” as the extent to which teachers and administrators in a school carefully and regularly look for problems, prevent problems from becoming crises, are reluctant to oversimplify events, focus on teaching and learning, are resilient to problems, and defer to expertise. Therefore, we also hypothesized in this work that components of the organizing mindfulness could have a significant role for the school principal in increasing or limiting his/her perceived work discomfort.

## 2. Materials and Methods

### 2.1. Aims of the Study

(1) Monitor the stress levels of a large sample of principals belonging to different levels of Italian schools; (2) verify the incidence of specific pathologies associated with high levels of school stress; (3) evaluate the relationships between the perceived stress, work discomfort and dimensions of organizational mindfulness; (4) test the fit of a general path model illustrating the influence of the predictors on principals’ work discomfort; and (5) identify the role of organizing mindfulness on principal’s perceived work discomfort.

### 2.2. Participants

The study was voluntarily attended by 419 school principals (131 males and 288 females) with an average age of 53.93 (SD = 6.46). They were invited to participate by means of an email indicating the purpose of the study, declaring the guarantee of anonymity and the use of the data collected for scientific purposes only, and therefore were requested to fill in a questionnaire online. Approximately 2500 contact e-mails were sent, extracting the addresses from a special national list. The sample size determination was made by setting a 1-alpha confidence level at 95%, therefore with z normal value at the confidence level of 1.96. The following formula was applied: X_ο_ = z^2^ (p × q)/b^2^, with p as the proportion to be estimated and q the proportion of complementary character and b the desired precision set at 5%. Hence: 3.8416 (0.31 × 0.69)/0.0025 = 329. The response rate recorded was 1:6, compatible with the fixed sample size (419 > 329). The average completion time was about 15 min. Tools administration took place upon the release and signing of the form for an informed consent of participation in accordance with the Declaration of Helsinki. The study was approved by the Institutional Review Board of the University of Cassino and Southern Lazio.

### 2.3. Tools

In order to collect the data necessary to carry out the study, a questionnaire was built up and articulated into the following sections: (1) Socio-demographic information: Gender; age; type of degree obtained; (2) school environment: (a) How long (in years) have you been a school principal? (b) How many students altogether attend the school(s) you currently manage? (c) How long (in years) have you been a principal at the current location? (d) type of school(s) related to the management (primary/secondary school, technical school, high school, etc.); (3) information on the current health of the subjects: Body mass index, high/low blood pressure, possible presence of diabetes, cholesterol, heart disease, respiratory problems (e.g., asthma, bronchitis), migraines, stomach problems, back, neck or joint pain; (4) psychometric measurements: (a) *The test M.S.P. (Psychological Stress Measurement)* [30,31]. The test measures the state of subjectively perceived stress, and consists of 49 items with Likert response scale 1–4 (from *not at all* to *very much*) on the individual’s perception of his cognitive-affective, physiological, and behavioral state. The overall test score provides a global index of the psychological stress state. In addition to the overall score, it is possible to calculate six other values that correspond to six different articulations of the way one perceives himself as being stressed: Loss of control and irritability (i.e., “I am irritable, my nerves are on edge, I lose patience with people and things.”); psychophysiological sensations (i.e., “I feel tense or strained.”); sense of effort and confusion (i.e., “I feel overwhelmed, overpowered and overloaded.”); depressive anxiety (mixed depression and anxiety symptoms: i.e., “I review the same ideas several times, I brood, I have the same thoughts over and over again, I feel my head full of thoughts.”); pain and physical problems (i.e., “I have physical pains: Back pain, headache, neck pain, bellyache.”); hyperactivity and acceleration (i.e., “I walk quickly”); (b) *Mindfulness Organizing Scale* (MOS) [32,33]. This is a self-report measure that investigates the safety of the organization, or rather how the worker perceives that safety. It is based on concrete behavior that reflects the employee’s relationship with the organization and his colleagues. The measurement is carried out on a three-point Likert scale (from *not at all* to *very much*). The dimensions included in the items are: Concern about failure, reluctance to simplify interpretations, sensitivity to operations, commitment to resilience, and deference to expertise. The S9, S5, and S3 scales were used for this study. A total of 9 items make up these three scales. The S9 scale measures the degree of application of group decision-making and comparison with colleagues (awareness of the value of shared problem analysis: i.e., “When a crisis occurs, we rapidly pool our collective expertise to attempt to resolve it”), the S5 scale is designed to assess the organization’s reluctance to simplify in the face of critical issues (awareness of the value of a non-rigid climate: i.e., “When discussing emerging problems with co-workers, we usually discuss what to look out for”), the S2 scale is intended to assess the level of organizational awareness (awareness of the value of mutual knowledge: i.e., “We discuss our unique skills with each other so that we know who has relevant specialized skills and knowledge”); (c) *Scheda per la rilevazione funzionale delle aziende* (Functional survey module for companies) [34]. For this study, within the module, the *Scale of Emotions at Work* was used; it consists of 10 items with true/false answers to assess the emotions that prevail during work. The direction of the scale is oriented to specifically assess the *Work Discomfort perceived* by the person; (d) *Polychronic-Monochronic Tendency Scale* (PMTS) [35]. This measures the subject’s ability to perform several tasks during the same time interval (i.e., “I feel at ease when I do several activities at once”). The measurement is carried out on a five-point Likert scale (from *strongly disagree* to *strongly agree*) including five items.

## 3. Statistical Analysis

The data were processed using the statistical software SPSS version 22 (IBM Corporation, Armonk, NY, USA) and Amos IBM version 22 (IBM Corporation, Armonk, NY, USA). The main analyses performed were: Descriptive statistics to illustrate socio-demographic information; Pearson and Spearman bivariate and partialized correlations for all main measures (Psychological Stress, Organizing Mindfulness, Work Discomfort, Polychronic-Monochronic Tendency) significant at *p* < 0.005 and at *p* < 0.001, 2-tailed); Kendal’s point-biserial correlations between MSP, Work Discomfort and reported physical ailments; Cronbach’s alpha as scale reliability coefficient; *T*-test to explore significance in Stress score and Polychronic Tendency relating to gender; Anova univariate test with *p* < 0.05 to explore significances between Work Discomfort, Stress and Organizing Mindfulness; hierarchical regression to identify the predictors of Work Discomfort and Stress; Cohen’s *d* and *Eta squared* as measures of effect size; SEM analysis to explore predictors’ effects on Work Discomfort. To test the adequacy of the model the following eight indices were considered: (1) the chi-square; (2) the relationship between the value of the chi-square and the degrees of freedom; (3) GFI (Goodness of Fit Index); (4) AGFI (Adjusted Goodness of Fit Index); (5) RMSEA (Root-Mean-Square Error of Approximation); (6) RMSR (Root Mean Square Residual); (7) CFI (Comparative Fit Index); (8) NFI (Normed Fit Index); (9) RFI (Relative Fit Index); (10) PNFI (Parsimony Adjustment to NFI); (11) PCFI (Parsimony Adjustment to CFI); (12) PCLOSE (testing the null hypothesis that the population RMSEA is no greater than 0.05).

## 4. Results

### 4.1. Descriptive Statistics

The main characteristics of the sample are illustrated in Table 1 below, while Table 2 presents the bivariate correlations between the measures used in the study. The corresponding dataset is available as Supplementary Material S1.

It can be observed in Table 2 that stress resulted inversely correlated to age, strongly correlated to work discomfort and inversely correlated to organizational awareness. Among the components of stress most associated with the perception of work discomfort were depressive anxiety (0.660 **), the sense of effort and confusion (0.601 **), and irritability (0.503 **). As the number of years of service increases, organizational awareness also improves (141 **), while stress (−0.173 **) and the perception of work discomfort (−0.162 **) decrease; at the same time, awareness of the value of mutual knowledge increases (0.149 **) and the person’s level of hyperactivity decreases (−0.150 **). The level of discomfort and perceived stress was not associated with the number of students in the administered institution. As seniority increased, there was also an increase in the number of students and therefore in the size of the school administered (0.138 **).

Partialized correlations with the perception of work discomfort, showed a decrease in the association between the variables (determined by the control variables), which however remained statistically significant with depressive anxiety (0.333 **), the sense of effort and confusion (0.264 **), awareness of sharing analysis (0.151 **), but no longer with irritability (0.041), awareness of no-stiffness (0.144), awareness of mutual knowledge (0.337).

Partialized correlations with the general measure of stress (MSP) showed a reduction of associations with age variables (−0.125 **) and work discomfort (0.098 **), and non-significance with awareness of sharing analysis (0.061), awareness of no-stiffness (0.008), awareness of mutual knowledge (−0.028).

Table 3 shows the distribution of stress levels among the sample, after the transformation of the raw score into T points and the relative comparison with the Italian percentile calibration values of the scale. It can be noted that 27.7% had high levels of stress and almost 60% of the principals were affected by moderate and high levels of stress.

**Figure 1:**
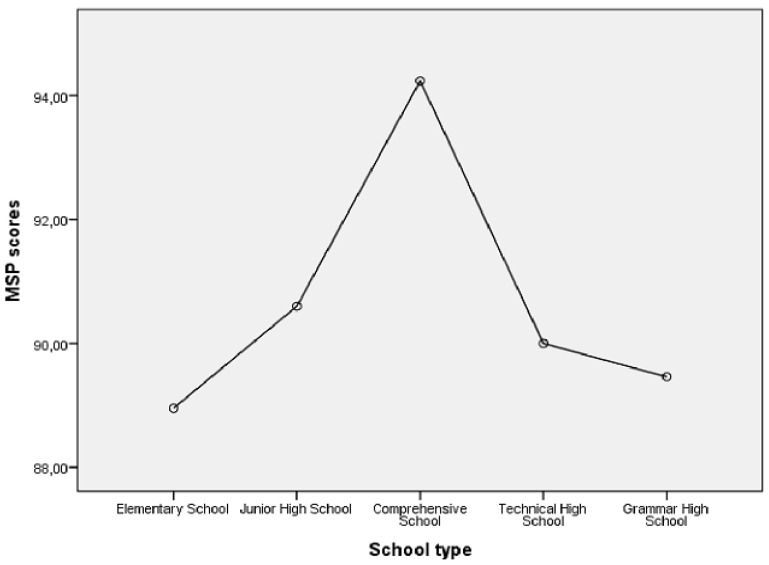
Type of School and Stress for the Principal.

Figure 1 reports the distribution of stress scores compared to the type of school managed. It can be observed that the regency of the Comprehensive Schools with multiple age levels (5–13) was accompanied by the highest level of stress for the principals, although the univariate ANOVA and Tukey’s post-hoc comparisons did not show significant differences between this level of stress and those associated with the other four types of school (*p* = 0.415).

Considering the gender of the principals, although there were no significant differences in the measure of perceived work discomfort (*p* = 0.131), the females reported a higher stress score: t (417) = −6637 *p* < 0.001 Sig: 0.000 M_1_ = 81.35 (DS = 17.83) M_2_ = 97.01 (DS = 24.17) 95% CI [−20.30; −11.02], *d* = 0.74; and a higher propensity, compared to males, to perform several tasks in the same time interval: t (417) = −3278 *p* < 0.001 Sig: 0.001 M_1_ = 2.86 (DS = 0.89) M_2_ = 3.16 (DS = 0.83) 95% CI [−0.470; −0.119], *d* = 0.42.

Univariate Anova reported, as illustrated in Table 4, a significant inverse association between *Organizational Mindfulness* and *Stress* scores; in addition, there was a significant association of the two variables (Stress and Organizational Mindfulness) with the measure of perceived *Work Discomfort*.

### 4.2. Stress and Principals’ Health

Table 5 reports point-biserial correlations between MSP, Work Discomfort and the physical ailments the principals claimed to have. It can be noted that the disorders most associated with stress and the perception of work discomfort are: Migraine, stomach problems, back and/or cervical pain. Stomach problems are sometimes also associated with respiratory problems (e.g., asthma, bronchitis).

### 4.3. Predictors of Work Discomfort

In order to identify among the components of stress and mindfulness the predictors influencing work discomfort perceived by principals, a hierarchical regression analysis was carried out. Hierarchical multiple regressions were run to determine if the addition of the Stress and Mindfulness components improved the prediction of Work Discomfort.

The preliminary verifications of the regression assumptions excluded the presence of multivariate outliers. Mardia’s multivariate kurtosis index (160.51) was in fact below the critical value [p (p + 2) = 168]; therefore, the relationship between the variables can be considered substantially linear. Low co-linearity was indicated by the low VIF values (Variance Inflation Factor) < 2 and high tolerance values > 0.60. For verification of the assumptions on the residuals, the average between the standardized and raw residuals was equal to 0; the Durbin-Watson test had a value of 1.97 and was therefore indicative of the absence of autocorrelation. Influential predictors have been identified in *Depressive Anxiety* (β = 0.426; ΔR^2^ = 0.439), *Sense of Effort and Confusion* (β = 0.259; ΔR^2^ = 0.033), and *Awareness of the Value of Shared Problem Analysis* (β = −0.255; ΔR^2^ = 0.034). The full model was statistically significant, *R*^2^ = 0.506, *F* (3, 418) = 41.975, *p* < 0.0005; adjusted *R*^2^ = 0.503.

### 4.4. Path Model

**Figure 2:**
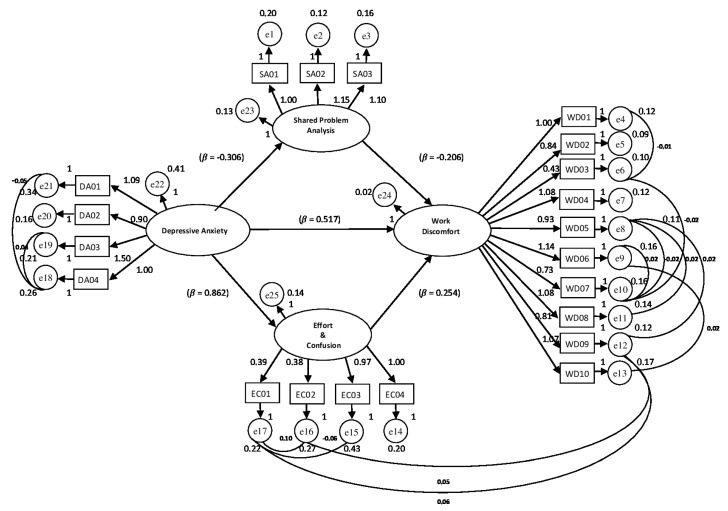
Path Model.

Subsequently, a SEM analysis was performed, combining into one explanatory model the variables that previously revealed significant association with Work Discomfort. The model showed overall good fit measurements: χ^2^ = 271.41 DF = 171 p = 0.000; CMIN/DF = 1.587; RMR = 0.016; GFI = 0.940; AGFI = 0.918. Baseline Comparisons NFI = 0.922; IFI = 0.970; CFI = 0.969. Parsimony-Adjusted Measures PNFI = 0.751; PCFI = 0.789; RMSEA: 0.037; PCLOSE: 0.995; RMSEA 90% 0.029–0.046. The model is displayed in Figure 2, where it is shown that *Work Discomfort* was mainly affected by *Depressive Anxiety* (standardized estimate of the regression weight of 0.517 for *p* < 0.018). The second influential predictor turned out to be the *Sense of Effort and Confusion* (standardized estimate of the regression weight of 0.862 for *p* < 0.001), which in turn receives a major influence precisely from *Depressive Anxiety* (standardized estimate of the regression weight of −0.248 for *p* < 0.010). The model has identified the *Awareness of the Value of Shared Problem Analysis* as a significant negative predictor of Work Discomfort (standardized estimates of the regression weights −0.206 for *p* < 0.001), furthermore this was negatively affected by *Depressive Anxiety* (standardized estimate of the regression weight of 0.517 for *p* < 0.018). Table 6 below summarizes the Maximum Likelihood Estimates and Regression Weights Estimates. The corresponding SEM with Amos is available as Supplementary Material S2.

## 5. Discussion

The state of chronic stress leads to an inability to manage events, both in the sense of not being able to solve the conditions of difficulty that arise and in the sense of inability to prevent them and even of unconscious tendency to intensify and proliferate obstacles and stressful events [36]. Numerous studies have demonstrated that chronic stress also directly produces innumerable illness conditions, influenced by the inability to manage and improve one’s own health [37].

Studies on work-related stress are increasing, as are publications on the difficult condition of school workers [38,39]. While the focus is on teachers, there are few reflections on the psychophysical fitness of the school manager, especially in light of the latest legislative and organizational changes. School leaders are also exposed to health risks related to work-related stress, but there are different aspects of the problem with respect to teachers and employees in general [40,41]. This involves an institutional figure thrown into the continuous proliferation of reforms, legal norms, structural changes, conflict management, and radical changes in customs and processes [42]. We are substantially dealing with a figure who is at risk and to whom more research attention should be paid for exploratory, diagnostic and preventive purposes.

Our study first of all showed that about half of the sample of principals who participated in the research had moderate to high stress levels. This immediately emphasizes how current and critical the problem is. The widespread tendency to merge different school cycles (elementary and Junior high schools) in the so-called “Istituti Comprensivi” (Comprehensive Schools, ages 5–13), for administrative reasons, was associated with a greater load of tension and pressure for principals, who probably find it difficult to manage with a single approach the problems and differences that arise from educational orientations and professional profiles traditionally characterized by a plurality of visions, different approaches to teaching and evaluation of students, diversity in the level of involvement of families in school life, different propensity and habit of teachers of different cycles (primary and secondary) to work on shared projects. It would seem that the management of these differences, rather than the size of the school (in terms of number of students, and therefore of teachers), is a reason for greater pressure and tension at work [43,44].

Women principals showed significantly higher levels of stress than their male colleagues. In this regard, according to the data, their greater tendency to engage simultaneously in the resolution of several tasks, could indicate a greater resistance to delegation, a strong sense of personal responsibility that would lead them to a total (psychological) involvement which over time can overload the person, limiting the time of physical and mental recovery. In line with these data, studies by Kiral [45] have shown that women principals have higher levels of stress than male principals, as women have to reconcile the responsibility for domestic work with the official and public work they do in school.

The first analyses of our study have indicated that the general perception of the principal’s working discomfort presents on the one hand an association with the level of stress, which contributes to increase the value of the discomfort, and on the other hand an equally significant association with organizing Mindfulness, which can substantially limit the negative effects of stress on perceived discomfort. This measure of discomfort includes de-motivational aspects, negative mood, disappointment for the unreliability of the context, negative balance between efforts and results, weight in conducting mediations, pessimistic view of the future, perception of others’ insensitivity, doubts about one’s self-efficacy.

An interesting reflection that emerged from the observation of the data was that relating to the age and period of service of the principals. Both the stress level and the perceived general discomfort had an inverse correlation with age and years of service. This suggests that experience can play a significant role in the development of the management and coordination skills required to best perform the complex functions of school leadership. The critical aspect concerns the Italian situation where in the last decade there has been a substantial turnover of principals. Therefore, only a few years ago, a large number of principals started their service role, in a context of extensive administrative changes imposed by the Ministry of Education. It is not difficult to hypothesize that in this situation the youngest principal may feel the weight and responsibility of an assignment that no longer finds a frame of reference in past experience.

The results of the study confirmed the association between high levels of stress and somatization, which was already evident in the literature on the health implications of a chronic occupational stress condition: Migraine, stomach problems, back and/or cervical pains, respiratory problems (e.g., asthma, bronchitis). These data were in line with Mariammal et al. [46] who stated that stress manifests itself in the form of chronic disorders or diseases such as hypertension, stroke, headache, and diabetes, as well as regular physical pain. Some principals experience symptoms such as suppression of the reproductive system, anxiety, aggression, indigestion, stomach-ache, pain, dizziness, and rapid heartbeat. In addition, chronic stress creates muscle tension, fatigue, constipation, and arthritis [47]. Principal stress has even been associated with severe problems such as ischemia and heart problems [48].

Further results of the study have identified the predictors of work-related discomfort with greater accuracy in the components of stress and organizational Mindfulness. Through a regression analysis and then through SEM, the effects and influence relationships between the predictors were identified. Among the components of the MSP, it seemed that depressive anxiety had the main role of influence. The anxiety component is characterized by aspects of recurrent ruminative cognition that amplify the sense of isolation, incomprehension, discouragement, and worry [49].

This negative interpretative framework activates another significant component: The sense of effort and confusion perceived by the person, who develops thoughts of inadequacy, the impression that everything involves a considerable effort and that everything falls on his shoulders. This attitude can naturally encourage a lack of clarity in ideas and decreased attention and concentration.

Within the model, one of the three components of mindfulness, the awareness of the importance of sharing problem analysis, found a significant place. The effect of the variable limited the dimension of the perceived discomfort. If, on the one hand, the increase in pressure and tension drives the person to intensify their efforts by closing and defensively stiffening themselves, on the other hand, the ability to recognize the value of sharing and involvement of other collaborators and colleagues in order to deepen the understanding of the problems and the identification of the most appropriate management methods, can help one to come out of isolation and discover the value of confrontation and the functional exercise of delegation, reducing the sense of oppression and distrust.

The anxiety component that characterizes the principal’s stress can be mitigated by training and refresher courses focused on a model of organizing Mindfulness. Acquiring awareness of the value of sharing practice is the main theme, but two other aspects to be investigated should not be underestimated and which in this study have however shown strong positive correlations with the practice of sharing, and which perhaps constitute the necessary operational preparatory basis: Awareness of the value/advantage of a non-rigid climate and awareness of the value of mutual knowledge.

A lack of awareness of one’s own way of acting can lead to behaviors that are not functional to the work context, to the quality of the interaction with one’s colleagues and to the nature of the task required at the time [50]. By investing in one’s mindfulness, one can expect to break the old automatisms in favor of new behaviors, effective even in difficult times, as indicated by Weick and Sutcliffe, who believe it is necessary to rely on a mindfulness-oriented approach when there is a need to make a quick and important decision, giving priority to one’s competence (or that of one’s co-workers), rather than relying on one’s authority.

The study by Beausaert et al. [51] emphasized the influence of social support in the containment of stress and the burnout of principals. Our study points out as a priority, above all, the awareness to which the principal must be individually accountable, i.e., the indispensability of a practice of sharing and mutual recognition of specificities and competences. In this way they move on to a proactive attitude that promotes social support in the first person, before expecting it (in due form) from others.

When Mindfulness becomes a shared social practice in an organization and permeates the routines, processes and practices among people and teams, and thus affects the organization as a whole, the organization itself becomes more resilient and proactive. Even the educational institution has a vital need to promote responsible leaders at all levels, capable of maintaining self-control, a sense of balance and self-determination, despite the informational overload they have to deal with today.

### Study Limitations

Data collection through self-reporting measures should be expanded with a methodological design in which judgments about principal stress could be provided also by the staff and teachers of the school, in order to have a more balanced representation by an outside perspective. The cross-sectional approach of the study involved collecting data at a single point in time; instead, the extension of the study could include repeated administration at different times of the school year (beginning, middle, end). The reference hypothesis is that the level of stress and the perception of discomfort could vary in relation to different significant institutional moments, such as the opening and closing of the school year. In this regard it could be useful the novel use of neuroscience-based approaches in education, namely neurodicatics, which is directed to address the educational and psychological well-being of students and staff involved in education as part of the education environment [52]. A further important contribution could be a specific focus on how principals cope with emergencies and on the functionality of their strategies to manage individual and collective stress triggered by the exceptional nature of the problems that the situation entails (e.g., ensuring teaching activities in safe environments after the spread of the Covid-19 virus). At the moment, there are also no longitudinal studies that have monitored the evolution of the principal’s leadership ability over medium-long intervals. It would therefore be important to understand if and how more mature and functional patterns for the containment and management of pressure in moments of personal tension and discomfort are learned and modeled over the course of his/her career.

## 6. Conclusions

The results of this study have shown that the levels of stress and work discomfort perceived by principals are high and require both empirical investigation and targeted support and prevention interventions. In fact, the stress experienced by a principal is associated with various physical disorders and serious health risks, such as ischemia and heart problems. The study has produced a path analysis model in which the joint effects between the main predictors of principals’ work discomfort were explained. The effect of depressive anxiety on perceived discomfort found a protective mediator in the mindfulness component that recognizes sharing as a fundamental operational tool, while an increasing sense of effort and confusion could significantly amplify the experience of psychological discomfort associated with the practice of school leadership.

## Figures and Tables

**Table 1:** Sample characteristics.

Gender	*n* (%)
Male	131 (31.3%)
Female	288 (68.7%)
**Geographical Area**	
Northern Italy	193 (46.1%)
Central Italy	103 (24.6.7%)
Southern Italy	123 (29.4%)
**Academic degree**	
Humanities	280 (66.8%)
Science	98 (23.4%)
Legal-Economic Disciplines	41 (9.8%)
**Years of service as principal**	M = 8.41 (SD = 7.22) Min = 1; Max = 38
**Years of service as principal in the current school**	M = 4.67 (SD = 4.23)
**School type**	
Elementary School (ages 5–11)	22 (5.3%)
Junior High School (ages 11–13)	10 (2.4%)
Comprehensive School (ages 5–13)	223 (53.2%)
Technical High School (ages 14–18)	86 (20.5%)
Grammar High School (ages 14–18)	78 (18.6%)
**Number of students per Institute**	M = 1.054 (SD = 437.22) Min = 50; Max = 3.600

Legend: M = Mean, SD = Standard Deviation; Min = Minimum; Max = Maximum.

**Table 2:** Bivariate correlations.

	AGE	MIND	MSP	WD	DA	IR	PS	SEC	DPF	HY	ANS	AMK	ASA	MPTS	SER	STU
AGE	1															
MIND	0.107 *	1														
MSP	−0.222 **	−0.244 **	1													
WD	−0.166 **	−0.379 **	0.652 **	1												
DA	−0.165 **	−0.291 **	0.878 **	0.660 **	1											
IR	−0.132 **	−0.177 **	0.743 **	0.503 **	0.679 **	1										
PS	−0.135 **	−0.162 **	0.725 **	0.371 **	0.614 **	0.569 **	1									
SEC	−0.139 **	−0.202 **	0.803 **	0.601 **	0.729 **	0.602 **	0.617 **	1								
PPP	−0.133 **	−0.172 **	0.641 **	0.365 **	0.566 **	0.463 **	0.574 **	0.485 **	1							
HY	−0.214 **	−0.092	0.568 **	0.261 **	0.429 **	0.464 **	0.412 **	0.406 **	0.365 **	1						
ANS	0.073	0.771 **	−0.192 **	−0.289 **	−0.204 **	−0.136 **	−0.105 *	−0.146 **	−0.132 **	−0.068	1					
AMK	0.122 *	0.870 **	−0.218 **	−0.344 **	−0.263 **	−0.165 **	−0.146 **	−0.156 **	−0.163 **	−0.107 *	0.526 **	1				
ASA	0.065	0.848 **	−0.189 **	−0.338 **	−0.253 **	−0.140 **	−0.149 **	−0.200 **	−0.133 **	−0.051	0.479 **	0.600 **	1			
MPTS	0.056	0.068	0.021	−0.087	−0.032	0.077	0.036	−0.091	0.050	0.234 **	0.020	0.038	0.104 *	1		
SER	0.593 **	0.141 **	−0.173 **	−0.162 **	−0.118 **	−0.080	−0.090	−0.118 *	−0.094	−0.150 **	0.095	0.149 **	0.083	0.017	1	
STU	0.046	0.017	−0.058	−0.060	−0.68	−0.093	−0.064	−0.072	−0.006	−0.039	0.023	0.004	0.031	0.078	0.138 **	1
*SKE (SE)*	−0.566 (0.119)	−0.560 (0.119)	0.828 (0.119)	0.922 (0.119)	0.844 (0.119)	10.15 (0.119)	10.28 (0.119)	0.971 (0.119)	10.12 (0.119)	0.322 (0.119)	−0.663 (0.119)	−0.302 (0.119)	−0.682 (0.119)	−0.017 (0.119)	10.13 (0.119)	20.52 (0.465)
*KUR (SE)*	−0.136 (0.238)	0.106 (0.238)	0.168 (0.238)	−0.065 (0.238)	0.110 (0.238)	10.19 (0.238)	10.22 (0.238)	0.638 (0.238)	0.683 (0.238)	−0.326 (0.238)	0.194 (0.238)	−0.315 (0.238)	−0.199 (0.238)	−0.102 (0.238)	0.974 (0.238)	10.52 (0.238)
*M (SD)*	540.93 (60.46)	20.41 (0.366)	920.11 (230.51)	20.52 (20.54)	10.81 (0.071)	10.66 (0.498)	10.40 (0.525)	10.66 (0.574)	10.71 (0.720)	20.16 (0.663)	20.48 (0.371)	20.21 (0.480)	20.52 (0.465)	30.06 (0.862)	80.41 70.22)	10540.25 (4370.21)
*alpha*	-	0.863	0.961	0.797	0.876	0.810	0.735	0.739	0.735	0.680	0.748	0.797	0.750	0.880	-	-

Legend: MIND = Organizing Mindfulness; MSP = *Psychological Stress Measurement*; WD = Work Discomfort; DA = Depressive Anxiety; IR = Irritability; PS = Psychophysiological Sensations; SEC = Sense of Effort and Confusion; PPP = Pains and Physical Problems; HY = Hyperactivity; ANS = Awareness of Non-Stiffness; ANK = Awareness of Mutual Knowledge; ASA = Awareness of Sharing Analysis; PMTS = Polychronic-Monochronic Tendency; SER = Years of Service; STU = Number of Students; M = Mean; SD = Standard Deviation; alpha = Cronbach’s alpha. N = 419; ** Correlation is significant at the 0.01 level (2-tailed). * Correlation is significant at the 0.05 level (2-tailed). For AGE, MSP, WD, SER, and STU Spearman’s correlation has been used.

**Table 3:** Distribution of stress levels in the sample.

Stress Level	*N*	%
Low (<25 percent.)	90	21.5
Medium (<51 percent.)	112	26.7
Moderate (<75 percent.)	101	24.1
High (>74 percent.)	116	27.7
Total	419	100.0

**Table 4:** Anova tests between Psychological Stress Measurement (MSP), work discomfort, and organizational mindfulness.

MSP							
Predictor	M*_Low_* (SD)	M*_High_* (SD)	F	*p* ^b^	Partial *η*^2^	90% CI	OP
**Organizing Mindfulness**	96.97 (1.57)	87.13 (1.61)	19.35	0.000	0.04	[89.84; 94.26]	0.98
**Work Discomfort**							
**MSP**	1.19 (0.14)	3.73 (0.36)	154.46	0.000	0.27	[2.26; 2.66]	0.99
**Organizing Mindfulness**	3.03 (0.14)	1.89 (0.25)	31.09	0.000	0.07	[2.26; 2.66]	0.99

Legend: M*_Low_* = Mean values of dependent variable in association with the lower values of predictor; M*_High_* = Mean values of dependent variable in association with the higher values of predictor; SD = Standard Deviation; OP = Observed Power. N = 419. Significance for *p* < 0.05. ^b^ = Adjustment for multiple comparisons: Bonferroni.

**Table 5:** Kendall’s Tau-b biserial point correlations between MSP, stress, and physical disorders.

	MSP	WD	HBP	DI	HC	HD	RP	MI	SP	BCP
MSP	1									
WD	0.502 **	1								
HBP	0.002	0.018	1							
DI	0.045	0.047	0.183 **	1						
HC	0.045	−0.003	0.058	0.180 **	1					
HD	0.069	0.044	0.250 **	0.034	0.142 **	1				
RP	0.089 *	0.072	0.063	−0.027	0.041	0.152 **	1			
MI	0.258 **	0.197 **	−0.074	−0.010	0.001	−0.056	0.086	1		
SP	0.268 **	0.185 **	0.051	0.032	0.100 *	0.087	0.171 **	0.183 **	1	
BCP	0.258 **	0.133 **	0.013	0.035	0.044	0.010	0.096	0.301 **	0.214 **	1

Legend: MSP = *Psychological Stress Measurement*; WD = Work Discomfort; HBP = High Blood Pressure; DI = Diabetes; HC = High Cholesterol; HD = Heart Diseases; RP = Respiratory Problems; MI = Migraine; SP = Stomach Problems; BCP = Back and/or Cervical Pain. N = 419; ** Correlation is significant at the 0.01 level (2-tailed). * Correlation is significant at the 0.05 level (2-tailed).

**Table 6:** Maximum likelihood estimates and standardized weight estimates.

Label		Label	Estimate	S.E.	C.R.	*p*	SWE
Depressive Anxiety	→	Shared Problem Analysis	−0.178	0.036	−4.957	***	−0.306
Depressive Anxiety	→	Effort and Confusion	0.991	0.065	15.248	***	0.862
Depressive Anxiety	→	Work Discomfort	0.198	0.047	4.198	***	0.517
Shared Problem Analysis	→	Work Discomfort	−0.136	0.034	−4.017	***	−0.206
Effort and Confusion	→	Work Discomfort	0.084	0.039	2.176	0.030	0.254

Note: *** *p* < 0.001.

## Supplementary Materials

The following are available online at https://www.mdpi.com/1660-4601/17/17/6318/s1, File S1: Principals Stress.sav (dataset); File S2: model work discomfort.amw (model).
